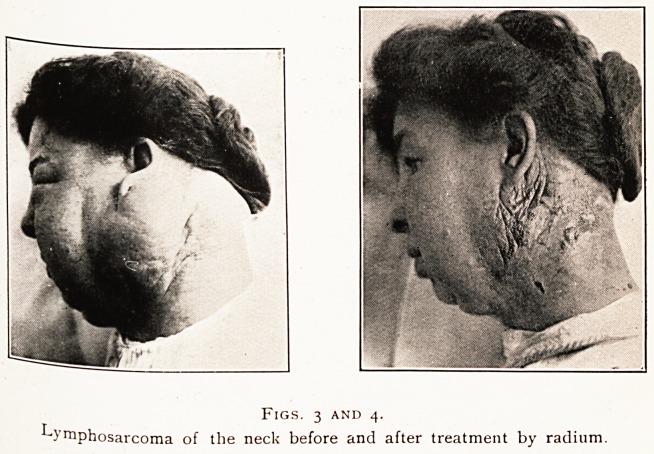# The Therapeutic Uses of Radium

**Published:** 1924-07

**Authors:** A. E. Hayward Pinch

**Affiliations:** Director of the Radium Institute, London


THE BRISTOL

MEDICO-CHIRURGICAL JOURNAL.

*"Scire est nescire, nisi id me
Scire alius sciret."*

JULY, 1924.

### THE THERAPEUTIC USES OF RADIUM.

BY

A. E. HAYWARD PINCH, F.R.C.S.,

*Director of the Radium Institute, London.*

---

I DESIRE first to express my appreciation of the compliment paid me in inviting me to lecture before the Bristol Medico-Chirurgical Society, and to voice the very keen and heartfelt pleasure I experience in meeting once again my former teachers and fellow-students. I spent six of the happiest years of my life at the old Bristol Medical School, first as student and later as Medical Tutor, and made many very real and lasting friendships, which I value more and more highly as the years roll on.

The subject of my address is "Radium Therapy," and as it is manifestly impossible to deal with it comprehensively in the time at my disposal, I propose to throw on the screen slides of patients suffering from various conditions which are amenable to radium, to say something of the history of the cases, and to indicate briefly the treatment which was adopted to produce the results shown.

Radium treatment may be carried out either with radium salt or radon (radium emanation) apparatus, their therapeutic properties being identical. At the Radium Institute itself radium salt apparatus is chiefly used; but when tubes or applicators are sent out of the Institute for employment in various parts of the British Isles the radon apparatus is supplied, the actual intrinsic value being so small that if mislaid or destroyed the pecuniary loss is but trifling. The tubes are charged with radium sulphate at least 90 per cent. pure, and there is no admixture with barium or any other inert material.

The flat surface applicators are made of three strengths: "Full strength," containing 0.5 centigram radium element to each square centimetre; "half strength," containing 0.25 centigram radium element to each square centimetre; "quarter strength," containing 0.125 centigram radium element to each square centimetre.

The strength of all radon (radium emanation) apparatus is expressed in millicuries, a millicurie being the amount of radon in equilibrium with a milligram of radium element.

When dealing with superficial or subcutaneous lesions the apparatus is employed either unscreened, or lightly screened, to cut out the soft and medium beta rays. The screens most generally used for this purpose are:—

Aluminium, 0.01 and 0.02 mm. thick.
Lead, 0.1 mm. thick.
Silver, 0.2 mm. and 1.0 mm. thick.

In the treatment of deeply-seated growths, or in cases where a prolonged radiation of *gamma* type only is necessary, a screen of lead 2.0 mm. in thickness is utilised, as such a screen practically cuts off all *beta* radiation.

When tubes are buried in tissues or tumours screens of 1.0 mm. of silver, 0.3 and 0.5 mm. platinum are the most generally useful.

I proceed now to place before you a series of lantern slides illustrating the results obtained by radium treatment in many and divers forms of disease.

### 1. LICHENIFICATION OF NECK.

The patient was a young married woman of thirty, an intensely neurotic subject, a nuisance to herself, a trial to her friends, and a curse to her husband.

The history of the case is most interesting, as it affords strong support to the theory that the affection is a neuro-dermatitis. The condition appeared about two weeks after the patient had sustained a severe nervous shock from seeing a child killed in a street accident. All remedies had failed to relieve the incessant and maddening irritation, and when the patient came to the Institute, the disease having then existed for six months, her mental condition was deplorable. She received one exposure of fifteen minutes' duration with "half strength" applicators screened with 0.01 mm. of aluminium. The irritation rapidly subsided, and within three weeks the skin was normal in character. Subsequently she had two recrudescences of the trouble. The first was determined by the shock of the loss of a relative on the *Titanic*, the second by an air-raid during the war. The same treatment was adopted on each occasion, with an equally successful result.

### 2. PSORIASIS.

The patient, a man of fifty, had suffered from the disease for fifteen years. He had received all kinds of treatment and occasionally experienced temporary benefit, but had never been cured. When first seen at the Radium Institute he had numerous large circinate patches over the trunk and upper extremities. He received three exposures, each of five minutes' duration, on three successive days with "half strength" applicators of appropriate shape and number. No screening was employed. All the lesions had cleared up within three weeks, and the patient remained free from any trouble for five years. At the end of that period he left London, and it has proved impossible to trace him.

This case is shown as a curiosity, as in the many other cases of psoriasis which I have treated recrudescence has generally taken place within six months.

### 3. LUPUS ERYTHEMATOSUS.

Patient a married woman of thirty-two. The disease had existed for seven years, and had proved most intractable, and resistant to all remedies, constitutional and local. The condition was of the "bat's wing" type, and very extensive, implicating the nose, both cheeks, and both lower eyelids. She was treated with "half strength" apparatus, screened with 0.01 mm. aluminium, the exposure being of forty-five minutes' duration. Great improvement followed, enabling the patient almost completely to hide the disfigurement by a slight application of face powder, and permitting her to take part in social functions. No cure of the condition was obtained, and exacerbations occur from time to time, but are always greatly benefited by the treatment above indicated.

### 4. LUPUS VULGARIS.

Patient a woman of fifty-six, an inmate of one of the London Poor Law Infirmaries. The disease had existed since childhood, was of a strongly ulcerative type, and had destroyed much of the nose and adjacent portions of the cheek. Scraping, cauterisation, X-rays and Finsen light had all proved ineffective. She was treated with "half strength" applicators unscreened, the exposure varying from one hour to one and a half hours' duration, repeated at intervals of six weeks over a period of six months. Complete healing resulted, and the disease was arrested. Patient was afterwards fitted with an artificial nose, and remained free from further trouble until her death six years later.

### 5. SENILE KERATOMATA.

Two patients, a man of sixty-four and a woman of seventy-two. Each patient had a large flat oval pigmented keratoma on a conspicuous portion of the face.

In each case an exposure of one and a half hours' duration with a suitable sized "half strength" applicator unscreened effected a complete disappearance of the lesion, leaving a smooth, supple and scarcely perceptible scar. There was no recurrence.

### 6. KELOID ACNE.

Patient a man of forty-two. Not a coloured individual. The disease had existed for nine years, and implicated the right half of hairy margin of scalp at the neck. Three exposures of one and a half hours' duration with "half strength" applicators unscreened were given at intervals of two months. Complete cure resulted.

### 7. KELOIDS.

One case of extensive degree, in a young woman of twenty-three, followed upon a petrol burn. The face and hands were very severely affected. Patient was compelled to wear a thick black veil when out of her home, and was scarcely able to use her hands. She was under treatment for two years. "Half strength" applicators, screened with 0.1 mm. of silver, were employed, and exposures varying from twelve to eighteen hours' duration given at intervals of six weeks. Improvement was slow, but steadily progressive, the keloidal tissue became absorbed, and the skin gradually assumed its normal appearances. At the present date scarcely any sign of the trouble is appreciable on the face or hands.

Another case, a schoolgirl of fourteen was playing in some charades, when some cotton wool which enveloped her neck and upper part of the chest was accidentally ignited. Keloidal changes occurred in the scar, drawing the chin down to the sternum, and preventing the patient from closing her mouth. A similar line of treatment was adopted and an equally successful result obtained.

It is advisable to treat keloids at as early a stage as possible, and before the bundles of collagen have developed into fully organised connective tissue. Special attention should be paid to the edges of the lesion, as increase proceeds by a peri-arterial fibrosis, which is most active at the periphery.

It is interesting to note that when once a keloid has received an adequate radiation all tendency to further growth is checked, though it may require several exposures to bring about a complete disappearance of the mass.

### 8. NÆVI.

(*a*) *Capillary Nævi ("Port Wine" Stains).*

Considerable improvement may often be effected in the appearance of patients disfigured by "port wine" stains of the face and neck, but the process is a tedious one and the exposures have to be very carefully calculated. It is never possible to restore the skin to its normal colour, but a very definite lightening of the tint may frequently be obtained, enabling the patient completely to hide the patch with a slight application of face powder.

The best results are usually obtained by exposures of from thirty to sixty minutes' duration with "half strength" applicators screened with 0.1 mm. of lead, three exposures being given on each of three successive days, and this series repeated at intervals of two months. The reaction should be of an intensity that produces desquamation without any vesication. The treatment is stopped when the discoloration is reduced to a salmon-pink tint.

(*b*) *Cavernous Nævi.*

The slides exhibited show very clearly the remarkably satisfactory results of radium radiation in the treatment of these growths.

In one case, a child of one year, a large oval pendulous growth, the size of a small cocoanut, implicated the lower half of the right cheek and the right sub-maxillary region. Its weight was such as to drag the child's head down to the right shoulder, and it was growing rapidly. Under appropriate treatment the nævus steadily decreased in size, and two years later it had completely disappeared, leaving only a very small flaccid area of skin over the right lower jaw.

The second case, an infant of five weeks, was brought with a very extensive prominent cavernous nævus implicating the lobe of left ear and the left pre-auricular region. Fifteen months later no traces of the growth remained.

The procedure adopted in both instances was "cross-fire" irradiation with "half strength" applicators screened with 0.1 mm. of lead, three exposures each of from twenty to fifty minutes' duration being given on three successive days, the series being repeated at intervals of six weeks.

The treatment of cavernous nævi with radium should be undertaken at the earliest possible opportunity, as infants prove much more susceptible than children approaching their 'teens, and if due care be taken no untoward result can occur.

### 9. RODENT ULCER.

Three distinct types may be recognised :—

(*a*) Lesions of the excavating type with thin overhanging edges and a soft gelatinous base, no rolled edge being present.

(*b*) Lesions of the hypertrophic and exuberant type, with slight superficial ulceration, and often limited by a distinct rolled edge.

(*c*) Lesions affecting the palpebral mucosa, outer and inner canthi.

Ulcers of the first group are the most difficult to treat satisfactorily, the absence of the rolled edge indicating that the patient's power of resistance to the disease is deficient and that no protective reaction is occurring. In these cases it is important to radiate some distance beyond the visible margins of the lesion, in order to deal with any outlaying invasive columns of cells which will almost certainly be present in those situations.

Repair in these cases is always slow, and sometimes imperfect, and they exhibit a very strong tendency to recurrence, so that they should be subjected to inspection at fairly frequent intervals, even if an apparent " cure " has been produced.

Ulcers of the second group usually do exceedingly well, and if the dose and exposure be correctly calculated one treatment may suffice to bring about an apparent cure. The tendency to recurrence in these instances is but slight, and many cases are recorded in the case sheets of the Radium Institute in which the patients have remained quite free from any recurrence since their treatment eight, ten or twelve years ago.

It is in the treatment of this particular class of lesion that the most striking, remarkable and permanent results of radium therapy are obtained. The complete disappearance of an extensive hypertrophic ulcerated mass, and its replacement by a smooth, soft, supple scar, differing but little from the surrounding skin, is often amazing, and might be regarded as incredible but for photographic evidence. In one of the slides shown an irregular hypertrophic ulcerated growth is seen to occupy the left side of nose and cheek and completely to occlude the eye. When the growth had disappeared under radium treatment the orbital fissure was found to be narrowed from cicatricial contraction of the affected eyelids, but the eye was quite healthy.

Rodent ulcer affecting the palpebral mucosa often responds well to radium treatment, which may completely arrest the disease, and prevent its extension to the sclerotic and cornea, and subsequent destruction of the eyeball. Many cases of this nature have been treated most successfully, the movements of the eyeball being unaffected and the vision unimpared.

Cases of rodent ulcer which have not previously received any active treatment with X-rays, ionization, $CO_2$ snow, etc., usually receive an unscreened exposure of from one and a half to two hours' duration, according to the thickness of the lesion, with a " full strength " applicator. The reaction is generally fairly severe, producing a degenerative necrosis, and removal by ulceration of all the malignant tissue, and possibly of a small surrounding layer of normal tissue.

The ulceration is followed by the formation of a " limpet shell " crust, underneath which repair occurs, leaving a smooth, soft, supple scar, particularly rich in elastic tissue fibres.

In cases of very long standing, accompanied by great destruction and invasion of the cartilaginous or bony tissues, unscreened exposures should not be given. Radium irradiation of any kind is powerless to cure rodent ulceration

FIGS. 1 AND 2.
Rodent ulcer of the orbit before and after treatment by radium.

FIGS. 3 AND 4.
Lymphosarcoma of the neck before and after treatment by radium.

of bone, and the same is almost equally true with respect to cartilage. For this reason the affected portions of these tissues should be removed as far as possible before radium treatment is commenced. A gamma radiation from heavily-screened apparatus applied to the borders of the growth and its deeper extensions will often serve to arrest the progress of the disease, diminish the pain, and induce some slight degree of repair, but it will not and cannot do more than this.

### 10. LYMPHADENOMA.

Patient a young girl of fifteen. The disease had developed with great rapidity. The glands of the left cervical region were principally affected, and in that situation they formed an enormous mass of firm, discrete, movable glands, obstructing the venous flow from the head and pressing upon the sympathetic; the patient's face was congested and dusky, and there was some respiratory embarrassment from displacement of the trachea.

Thirty hours' treatment was given with surface applicators containing 500 mg. of radium bromide, screened with 2 mm. of lead, applied so as to produce a "cross-fire" radiation. The glands rapidly decreased in size and number, and six weeks later were neither visible nor palpable, the patient being apparently in normal health.

She kept quite well for two years, and then went to Roumania. Shortly after her arrival in that country an acute exacerbation of the disease took place, affecting most of the superficial glands of the body. She was unable to obtain further radium or X-ray treatment, and the disease rapidly progressed to a fatal termination.

### 11. EPITHELIOMA.

Slides showing a series of recurrent epitheliomatous growths implicating the nose, scalp, and head.

In each instance the diagnosis had been established by microscopical examination and the primary growth excised. The patients had refused further operation. All the lesions affected the glabrous skin, and were of the rapidly fungating type. Treatment was carried out by the burying of many small tubes of radium screened with 0.3 mm. of platinum in the substance of the tumour, "cross-fire" radiation with unscreened "full strength" applicators applied to the surface of the growth for two to three hours, and a prolonged gamma radiation of the associated lymphatic area. "Apparent cure" was obtained in each case.

### 12. SPHEROIDAL-CELLED CARCINOMATA.

*Cancer of the breast.*—This series of slides indicates the results obtained in the treatment of inoperable or recurrent carcinoma of the breast. Absorption of nodules in the scar area, healing of ulceration, and diminution of lymphatic engorgement are all clearly shown. It is not possible to speak of "cure" in these conditions, but very real improvement in the local condition is often obtained, and the disease rendered quiescent for many years. At the present moment we have on the case sheets of the Radium Institute some half dozen or more patients who came for the treatment of extensive inoperable recurrence ten years ago. The treatment in these cases is carried out almost entirely by prolonged gamma radiation of thirty hours' duration with heavily screened applicators. In the case of small nodules in a readily accessible situation the insertion of small tubes of radium, screened with 1 mm. of silver, into their substance for twenty-four hours is often of great value.

### 13. SARCOMATA.

Two cases of periosteal sarcoma.

The first was a woman of sixty-two. She was treated in June, 1913, for a rapidly growing recurrence on the right clavicle after removal of the primary growth early in May, 1913. Berger's operation had been suggested as the only method of extirpating the disease, but the patient had declined. Microscopical examination of a portion of the original growth showed it to be a round-celled periosteal sarcoma. A tube containing 100 mg. of radium bromide, screened with 1 mm. of silver, was inserted into the tumour and left for twenty-four hours. The growth steadily shrank, and two months later no trace of it was appreciable. No recurrence has taken place, and the patient is alive to-day (March, 1924).

The second case was a young married woman of thirty-two. She was first seen in February, 1914. She then had a large fixed growth the size of a small cocoanut on the outer aspect of the upper third of the right thigh. Two attempts had been made to remove it by operation, but without success, and the tumour had grown rapidly. Amputation at the hip-joint had been suggested, but declined. The patient was unable to stand or walk, and the pain was so severe that she had to be kept constantly under the influence of opiates. Microscopical examination of portions removed at operation showed the tumour to be a spindle-celled periosteal sarcoma. The patient was treated twice, at intervals of six weeks, in February and April, 1914. Two tubes, each containing 100 mg. of radium bromide, and screened with 1 mm. of silver, were used on each occasion, one being buried in the upper and one in the lower half of the tumour. Rapid and steady improvement followed, the growth shrank, being converted into a dense fibrous nodule the size of a walnut, all pain disappeared, the patient speedily regaining her health and strength. No further treatment has proved necessary, and at the present date (March, 1924) the patient is in excellent health, walks, dances, cycles and swims perfectly, and is working eight and ten hours a day.

### 14. LYMPHOSARCOMATA.

The lymphosarcoma cell is particularly susceptible to gamma radiation, and its degeneration rapidly occurs.

The patient was a married woman of forty, and when seen at the Institute in 1913 she had an enormous lymphosarcomatous tumour in her left cervical region, spreading forwards over the descending ramus of the left jaw, and causing great œdema of the face, the left eye being completely closed.

The patient received a course of prolonged gamma radiation, applicators containing half a gramme of radium bromide, screened with 2 mm. of lead, being used, and an exposure of thirty hours' duration being given. Six weeks later the greater part of the tumour in the neck had disappeared, the œdema of the face had gone, and the left eye was no longer closed. The treatment was repeated, with the result that the growth practically vanished, and the patient regained her normal health. She kept well until July, 1915, when she was confined with twins. This was followed by a rapid recrudescence of the disease, numerous growths appearing in various parts of the body, and she died in November, 1915.

Three patients were exhibited, viz. :—

Mr. Hey Groves showed a female patient with a large-celled sarcoma of the neck and extending into the left upper clavicular fossa. During attempt at removal the left subclavian artery, being deeply involved in the growth, was torn and had to be ligated. It being impossible to make a complete removal of the neoplasm, an emanation tube (=9 millicuries) was inserted.

*Result.*—At present (six months later) there is no evidence of the growth.

Sections of the growth were exhibited showing characteristic round-celled sarcoma.

Dr. Patrick Watson-Williams showed a female patient, aged 52, in whom a sarcoma, a section of which showed it to be of the orbital type, had appeared intra-nasally in the left upper ethmoidal region and had caused a swelling in the left upper inner orbital angle, and (as seen in the photographs exhibited) with resulting slight displacement of the globe downwards and outwards, and consequent diplopia. The intranasal ethmoidal portion had caused epiphora, probably from involvement of the lachrymal duct. Mr. Hayward Pinch had seen the patient with him on February 29th, and very shortly after the earliest manifestations of the growth, and considered it favourable for treatment by radium. On March 4th two emanation tubes were inserted, the one intranasally being buried deeply in the ethmoidal cells, the other inserted a full inch deeply into the orbital swelling through an incision in the skin. Now, eight days later, all the diplopia and epiphora had disappeared, the eyeball being no longer displaced, though the swelling of the neoplasm in the orbital angle was still obvious. (This patient died on April 20th with extensive sarcomata of the lung and liver.)

Mr. Duncan Wood showed a female patient, aged 69.

*Symptoms.*—One year ago she noticed a small swelling in the middle line of her neck; it was not painful. Four months ago she had a boil behind her left ear. The lump in her neck then commenced to grow more rapidly, and became painful; the pain is worse on swallowing, but she is able to take any kind of food.

*Physical signs.*—There is a swelling in the position of the thyroid gland. It is hard, but its surface is smooth; it is fixed to the trachea, but moves on swallowing. No pressure signs on the vessels or nerves in the neck. The larynx is normal.

Mr. Hayward Pinch said that the fixity of the swelling to the trachea suggested that it was a malignant thyroid, but that its smooth surface was not typical, and that chronic thyroiditis had to be considered. He was strongly against surgical interference. He did not advise burying radium in the swelling, as in these cases there was a great tendency for a fungating mass to break through the skin. He would treat it by radiations through the skin. He thought that X-rays would be helpful.

### DISCUSSION.

Mr. Walters asked what was to be recommended in prostatic epithelioma recurring after operation, and whether radium was of value in carcinoma of the rectum.

Mr. Hayward Pinch said that if, in the case of malignant disease of the prostate, the growth was circumscribed and confined to one lobe, the best results were usually obtained by the burying of a tube of about 50 m.c. screened with 1 mm. of silver in the centre of the mass for twenty-four hours.

If the whole gland was affected, radiation should be given by means of a tube in the urethra, a tube in the rectum, and a plate over the perinæum.

In carcinoma of the rectum, fungating growths not completely encircling the canal, should be treated with emanation tubes screened with 1 mm. of silver and buried in the tumour for twenty-four hours. If the growth was of the annular type with much surrounding infiltration, a powerful tube screened with 2 mm. of lead should be placed within the lumen of the structure for twenty-four hours, supplemented by external radiation from a screened plate applied over the sacrum at the level of the growth.

Dr. Patrick Watson-Williams asked whether the suggestion that optic nerve atrophy might result from radium applied in the neighbourhood of the eye had any substantial foundation in Mr. Hayward Pinch's experience. He had been particularly interested in the case of rodent ulcer which, as shown in the slide exhibited, had become so extensive and advanced that the eyeball was completely buried.

In this case Mr. Hayward Pinch said that, believing the eye had already been destroyed, he had given very heavy radium exposures, with the result that the malignant growth completely disappeared, bringing to view and to restored functional activity the long-buried eye.

Mr. Cyril Walker referred to a question that had been put by the late Dr. Stack, viz. what ocular precautions were required against radium ? He cited a case of irritation of the eyes following X-rays for odontograms.

Mr. Hayward Pinch replied that from his experience of two thousand cases in which exposures of from fifty minutes unscreened to thirty hours heavily screened had been made he did not think it possible that radium could cause atrophy of the nerve or injury to the sight ; at any rate, he had never seen any papillitis in his cases. In reference to a question from another speaker he said that for epithelioma of the cornea he recommended and had used exposures of fifty minutes, unscreened.

As regards carcinoma of the cervix, in reply to Dr. Statham, he stated that if the broad ligament was involved, no cure could be expected, but for uncomplicated cervical epithelioma he would remove and follow up with radium.

Dr. Bartholomew asked whether radium was of value in mycosis fungoides.

Mr. Hayward Pinch replied he had had good results. In reference to radium in tumours of the brain he had had five cases, and referred to one seen with Sir Charles Ballance, in which three exposures totalling 20,000 milligram hours were given after decompression, and now three years later the patient was alive.

Dr. J. A. Nixon spoke of the beneficial results in his experience from use of radium in lupus erythematosus.

Dr. Odery Symes referred to a case of carcinoma of the œsophagus and of the wonderful relief following radium, the patient, who had been unable to swallow anything but fluids, being enabled to eat and enjoy any ordinary solid meal. Yet after an interval of twelve months the growth recurred and caused œsophageal obstruction, and the second radium application failed to afford relief.

Mr. Hayward Pinch emphasised the importance of early radium treatment in these cases, as when the lumen of the œsophagus was obliterated the radium could not be introduced and so could not reach well into the centre of the growth, which was so essential. He showed his newly-designed radium applicator, which being "hour-glass" shaped could be inserted and left attached by a thread, as the shape of the shield retained it *in situ* without the necessity of a long stiff retaining stem extending from the mouth to the tube. The patient could thus tolerate the tube and retain it *in situ* as many hours as required, when by pulling on the thread the applicator could be withdrawn.

## Figures and Tables

**Figs. 1 and 2. f1:**
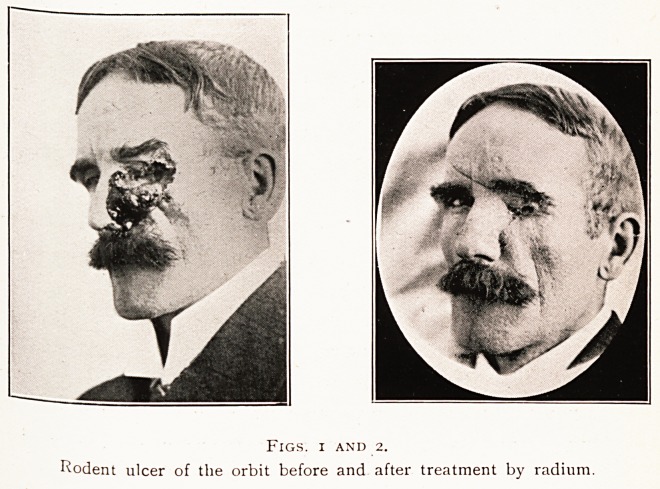


**Figs. 3 and 4. f2:**